# Genome sequence of *Staphylococcus epidermidis* H1G7 from hilsa (*Tenualosa ilisha*) gut

**DOI:** 10.1128/mra.00669-26

**Published:** 2026-09-16

**Authors:** Fahim Al Galib, Abdullah Al Kafi, Dipali Rani Gupta, Tahsin Islam Sakif, Morshedur Rahman, Md. Shahjahan, Md. Mahbubur Rahman, Md. Golzar Hossain, Tofazzal Islam

**Affiliations:** 1 Institute of Biotechnology and Genetic Engineering (IBGE), Gazipur Agricultural University (GAU) 198780, Gazipur, Bangladesh; 2 West Virginia University 5631, Morgantown, West Virginia, USA; 3 Department of Dairy and Poultry Science, Gazipur Agricultural University (GAU) 198780, Gazipur, Bangladesh.; 4 Department of Fisheries Management, Bangladesh Agricultural University 54492, Mymensingh, Bangladesh; 5 Department of Microbiology and Hygiene, Bangladesh Agricultural University 54492, Mymensingh, Bangladesh; Montana State University, Bozeman, Montana, USA

**Keywords:** cyclodipeptide synthase, gut bacterium, metal-chelate transport, fish probiotic, short chain fatty acid (SCFA) genes

## Abstract

We report the genome of *Staphylococcus epidermidis* strain H1G7 from hilsa (*Tenualosa Ilisha*) fish gut. Nanopore sequencing produced a 2.5-Mb assembly (32% GC, two contigs). The genome encodes metal–chelate transport, short-chain fatty acid production, and biosynthetic clusters and lacks virulent factors, revealing genomic features potentially associated with beneficial host interactions.

## ANNOUNCEMENT

*Staphylococcus epidermidis* is a ram-positive, facultative anaerobic, coagulase-negative bacterium that functions as a dominant commensal of the human skin microbiome and a potential probiotic while also acting as an opportunistic pathogen in mammals, including humans (1, 2). In our previous culture-independent 16S rRNA gene-based microbiome studies, members of the genus *Staphylococcus* were consistently detected in the gut microbiota of wild hilsa (3, 4).

These observations prompted us to investigate culturable *Staphylococcus* populations in hilsa. This led to the isolation of strain H1G7 from the gut of hilsa shad (*Tenualosa ilisha*), Bangladesh’s national fish, captured by local fishermen using gill nets from the Meghna River in Chandpur, Bangladesh (22.8333°N, 90.8333°E). Fish were processed immediately after transport on ice. The gastrointestinal tract was aseptically removed and homogenized in 9 mL phosphate-buffered saline (PBS, pH 7.0) and plated (serially diluted 1–1 × 10^−7^) on mannitol salt agar (Liofilchem, Italy) (32°C for 48 h) (5). Distinct 15 colonies were purified by repeated streaking. A single colony designated as H1G7 exhibited mannitol fermentation (yellow zone) and positive catalase activity.

Genomic DNA from an overnight same medium pure culture was extracted using the FavorPrep Tissue Genomic DNA Extraction HE Mini Kit (Favorgen, Taiwan) and quantified with a Qubit 4.0 fluorometer (Thermo Fisher Scientific).

Libraries were prepared using the Oxford Nanopore Technologies (ONT) Ligation Sequencing Kit SQK-NBD114.96 with the NEBNext companion module (New England Biolabs) and Native Barcoding Kit 96 (v14) with no DNA shearing and no size selection and sequenced on an ONT MinION using an R10.4.1 flow cell (6). Basecalling with Dorado v1.2.0 (SUP model, Phred >7) yielded 50,639 reads totaling 396,453,637 bp (*N*_50_, 11,797). Adapter sequences were trimmed using Porechop v0.2.4 (7). Reads were quality-assessed using NanoPlot v1.46.1 (8), *de novo* assembled with Flye v2.9.5-b1801 (9), and polished with Medaka v2.0.1 (10). The final assembly quality and completeness were assessed using QUAST v5.2.0 (11) and CheckM v1.2.4 (12). The genome assembly was annotated using the National Center for Biotechnology Information Prokaryotic Genome Annotation Pipeline (NCBI PGAP) v6.10 (13). Default parameters were applied for all software, unless otherwise specified.

The gapless genome (retain unrotated) comprised one complete circular chromosome and one plasmid, with a total size of 2.52 Mb (32% GC; 69× coverage), encoding 2,436 genes (2,355 protein-coding, 61 tRNA), 69 pseudogenes, and 99.4% completeness with no detected contamination (Table 1). GTDB-Tk v2.6.1 (14) assigned H1G7 to *S. epidermidis* (GCA_900458605.1) with 97.14% average nucleotide identity (ANI) (~90% coverage).

**TABLE 1:** Genomic features of *S. epidermidis* H1G7 isolated from gut content of hilsa

Genome feature	H1G7
GenBank	JBSVSH010000000
Assembly	GCA_054403095.1
SRA	SRS28386803
BioSample	SAMN53736889
Number of reads	50,639 (*N*_50:_11,797)
Assembled genome size (bp)	2,522,553 bp
GC content (%)	32%
Coverage (×)	69×
Genome completeness (%)	99.4% (CheckM)
Contamination (%)	0%
*N*_50_ value (bp)	2,509,976
Number of contigs	2
Genes (total)	2,436
Coding sequences (CDSs)	2,355
Genes (coding)	2,296
CDSs (with protein)	2,296
Genes (RNA)	81
rRNAs	6, 5, 5 (5S, 16S, 23S)
Complete rRNAs	6, 5, 5 (5S, 16S, 23S)
tRNAs	61
ncRNAs	4
Pseudo genes	69
CDSs (without protein)	69
Pseudo genes (ambiguous residues)	0
Pseudo genes (frameshifted)	33
Pseudo genes (incomplete)	28
Pseudo genes (internal stop)	24
Pseudo genes (multiple problems)	19
CRISPR arrays	2
Plasmid replicon	1

Genomic analysis (gutSMASH v2.0.1; 15) predicted short-chain fatty acid (SCFA) production and polyamine biosynthesis linked to *Klebsiella* and *Salmonella* inhibition (16), as well as intestinal integrity support, inflammation reduction, and host growth and immunity enhancement (17, 18). Seven biosynthetic gene clusters detected via antiSMASH 8.0.4, including NRPS-derived cyclic depsipeptides (icosalide A/B), RiPPs, and betalactones, contributed to antimicrobial activity and niche adaptation (19, 20).

Notably, no antimicrobial resistance genes were detected (ResFinder v4.7.2; 21), and the genome showed low pathogenic potential (score 0.48; PathogenFinder v2.0.6; 22). The genome encodes CntABCDF metal-chelate transport and a cyclodipeptide synthase for iron-chelating pigment production (23) and provides a first resource for further investigation H1G7 as a potential probiotic candidate.

## Data Availability

The whole-genome shotgun project of the *S. epidermidis* strain H1G7 has been deposited in GenBank assembly under accession number GCA_054403095.1 within BioProject (Mining Bangladesh Biogold) accession number PRJNA1055760. The version described in this paper is version JBSVSH000000000.
